# Nanoscale Au–Si eutectic mixtures formed by dewetting of a Au–Ni film on Si_3_N_4_

**DOI:** 10.1039/d5ra04389a

**Published:** 2025-10-31

**Authors:** Yoonhee Kim, Kang Woo Ahn, Su Yong Lee, Jin Woo Kim, Hyon Chol Kang, Chan Kim, Do Young Noh

**Affiliations:** a European X-ray Free-Electron Laser Facility Holzkoppel 4 Schenefeld 22869 Germany yoonhee.kim@xfel.eu chan.kim@xfel.eu; b Department of Physics and Photon Science, School of Materials Science and Engineering, Gwangju Institute of Science and Technology Gwangju 61005 Korea dynoh@gist.ac.kr; c Pohang Accelerator Laboratory Pohang 37673 Korea; d Department of Materials Science and Engineering, Chosun University Gwangju 61452 Korea

## Abstract

Formation of a eutectic AuSi compound was observed during the solid state dewetting and agglomeration in a Ni–Au bilayer thin film grown on a Si_3_N_4_ substrate using coherent X-ray diffractive imaging, X-ray diffraction, and scanning electron microscopy. During rapid thermal annealing at 850 °C in vacuum, AuNi films first separate into islands that are composed of a mixture of Au-rich and Ni-rich phases. As the dewetting proceeds, Si and nitrogen dissociate due to the catalytic action of Ni, which resulted in the formation of NiN, NiSi, and AuSi. With increasing time of the annealing process, nitrogen atoms in NiN are gradually evaporated by forming N_2_. The eutectic phenomenon in AuSi alloys results in the migration of Au atoms to form Au_5_Si_2_ with a composition near the eutectic point, 18.6 at% of Si. Our findings indicate that nucleation of the Au_5_Si_2_ alloy formation does not primarily occur through bulk interdiffusion, but instead initiates through grain boundary diffusion of Au atoms.

## Abstract 2

Formation of a eutectic AuSi compound was observed during the solid state dewetting and agglomeration in a Ni–Au bilayer thin film grown on a Si_3_N_4_ substrate using coherent X-ray diffractive imaging, X-ray diffraction, and SEM.

## Graphical abstract

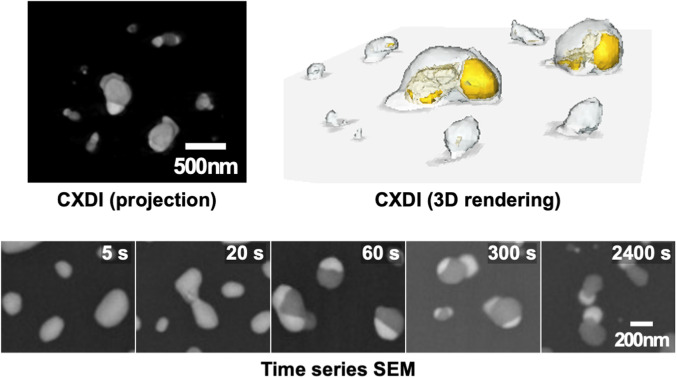

## 1 Introduction

Intermetallic compound systems are of great interest due to their excellent physicochemical properties and wide range of applications, *e.g.* in petroleum-refining, steel, and aeronautics industries. AuNi alloys, in particular, are one of the most commonly used alloy systems due to their simple phase diagram and attractive functionalities, *e.g.*, aging and superparamagnetic behaviors.^1–3^ Although bulk Au–Ni systems are immiscible at low temperatures due to limited solubility to each other, the solubility increases at elevated temperatures resulting in intermediate Au- and Ni-rich phases. In thin Au–Ni films, due to the large lattice mismatch between Au and Ni (Δ*a*/*a* ∼15%),^4^ intermediate mixed phases of various compositions form and the atomic relaxation plays a critical role in solid-state dewetting and agglomeration. AuNi bilayer films, widely employed in thin film devices like laser diodes and light emitting diodes, have been investigated as a model system for bilayer dewetting processes.^5^

In recent years, the importance of crystal shape, domain structure, and their atomic composition to physicochemical properties of intermetallic systems has been addressed.^6,7^ Also, the controlling of domain structure, *e.g.* the atomic ordering and spatial arrangement of the different kinds of atoms, becomes one of crucial parameters in improving the properties of intermetallic compounds.^6^ The atomic composition and ordering information are acquirable with high sensitivity by typical X-ray diffraction (XRD) techniques including nanobeam XRD, but they can only provide overall information of sample illuminated with the X-ray beam.^5,8,9^ Atomic resolution imaging on surface sensitive structures or sliced samples is available by electron microscopes, but an inherent limitation caused by the short mean free path of electrons makes comprehensive three-dimensional imaging impractical. 3D coherent X-ray diffractive imaging (CXDI) method,^10–15^ which is capable of quantitative nondestructive imaging, can unveil crucial missing information such as inner or buried structures although its practical spacial resolution is still limited to a few nm.^16–18^

Dewetting and agglomeration phenomena are strongly related to the minimization of the surface free energy and the increase in surface-to-volume ratio.^19–24^ These phenomena are widely utilized in the fabrication of nanoparticles of desirable functions,^22,24,25^ but often lead to a failure of thin film devices.^20,26^ When the supporting substrate of a bilayer film is involved in the reaction, the dewetting and agglomeration behavior becomes more complicated. Since the interaction with the substrate materials can trigger dewetting and formation of complicated compounds, it is essential to understand the interaction to fabricate high quality devices and nanoparticles.

In this paper, we present a nondestructive 3D visualization of the formation of eutectic AuSi compounds during solid state dewetting and agglomeration in a Ni–Au bilayer thin film grown on a Si_3_N_4_ substrate. CXDI method is applied for non-destructive 3D visualization of nanoalloy particles, and XRD and scanning electron microscope (SEM) measurements are conducted to investigate the resulted phenomenon in a time series manner. Atomic mixing near the interfaces forms Au_3_Ni and AuNi_3_ compounds, NiSi and NiN alloys in the beginning, and eutectic behavior of AuSi alloys triggers atomic diffusion along domain boundaries^19^ followed by formation of metastable Au_5_Si_2_ and Ni_3_Si_2_ alloys.

## 2 Experimental details

The tomographic CXDI experiment was conducted at a coherent X-ray scattering beamline (9C beamline) at Pohang Light Source-II (PLS-II) in Korea. The X-ray energy was fixed at 6 keV (*λ* = 2.07 Å) by a silicon double crystal monochromator (DCM) and the second crystal was detuned to filter the higher harmonics. The X-ray beam was focused by a rhodium-coated silicon toroidal mirror to maximize the coherent flux of the incident X-ray beam on a sample and eliminate the higher harmonics.^27,28^ A rhodium-coated silicon toroidal mirror was employed to maximize the flux density of the X-ray beam and eliminate the higher harmonics.^27,28^ A beam defining tungsten (W) pinhole with 10 μm diameter was placed near the focus to have enough coherency at the interaction plane, and two sets of beam cleaning silicon slits were placed in front of the sample. The diffraction patterns of the phase separated AuNi nanoalloys were measured by a PILATUS 1M detector with 172 μm pixel size. The PILATUS detector was placed 5 m downstream from the sample and a beamstop was used to protect the detector from the direct X-ray beam.^29^

Phase-separated AuNi nanoalloys including the eutectic AuSi compound were fabricated by using the solid state dewetting and agglomeration processes in a pre-shaped Ni–Au bilayer film on a Si_3_N_4_ substrate. We used e-beam lithography to pattern a 1.5 μm disk on 100 nm thick Si_3_N_4_ membrane window, and sequentially deposited each of 10 nm thick Ni and Au films by e-beam deposition. The Ni–Au bilayer film thus prepared was annealed at 850 °C in vacuum (∼10^−6^ mbar) for 20 min to convert it to phase-separated AuNi nanoalloy particles including the eutectic AuSi compound. The ramp-up rate was 200 °C min^−1^ during the heating process and the temperature dropped to 300 °C in less than a minute during the cooling process.

In addition, a series of samples with various annealing times, from 1 s to 3600 s, were prepared to investigate time dependence of the phase behavior. We first deposited each of contiuous 10 nm thick Ni and Au films on 100 nm thick Si_3_N_4_ layer supported by thick Si(100) substrate using e-beam deposition. Then, the Ni–Au bilayer films were annealed in exactly the same procedure as described previously with various annealing time. XRD measurements on the prepared samples were carried out at GIST X-ray scattering beamline (5D beamline) at PLS-II in Korea. A 10 keV (*λ* = 1.24 Å) X-ray was selected by using a silicon DCM and focused by using a cylindrical X-ray mirror and a sagittally bent monochromator in the vertical and horizontal directions respectively.^27,28^

## 3 Results and discussion

### 3.1 Three-dimensional visualization of the eutectic AuSi compounds

**Fig. 1:**
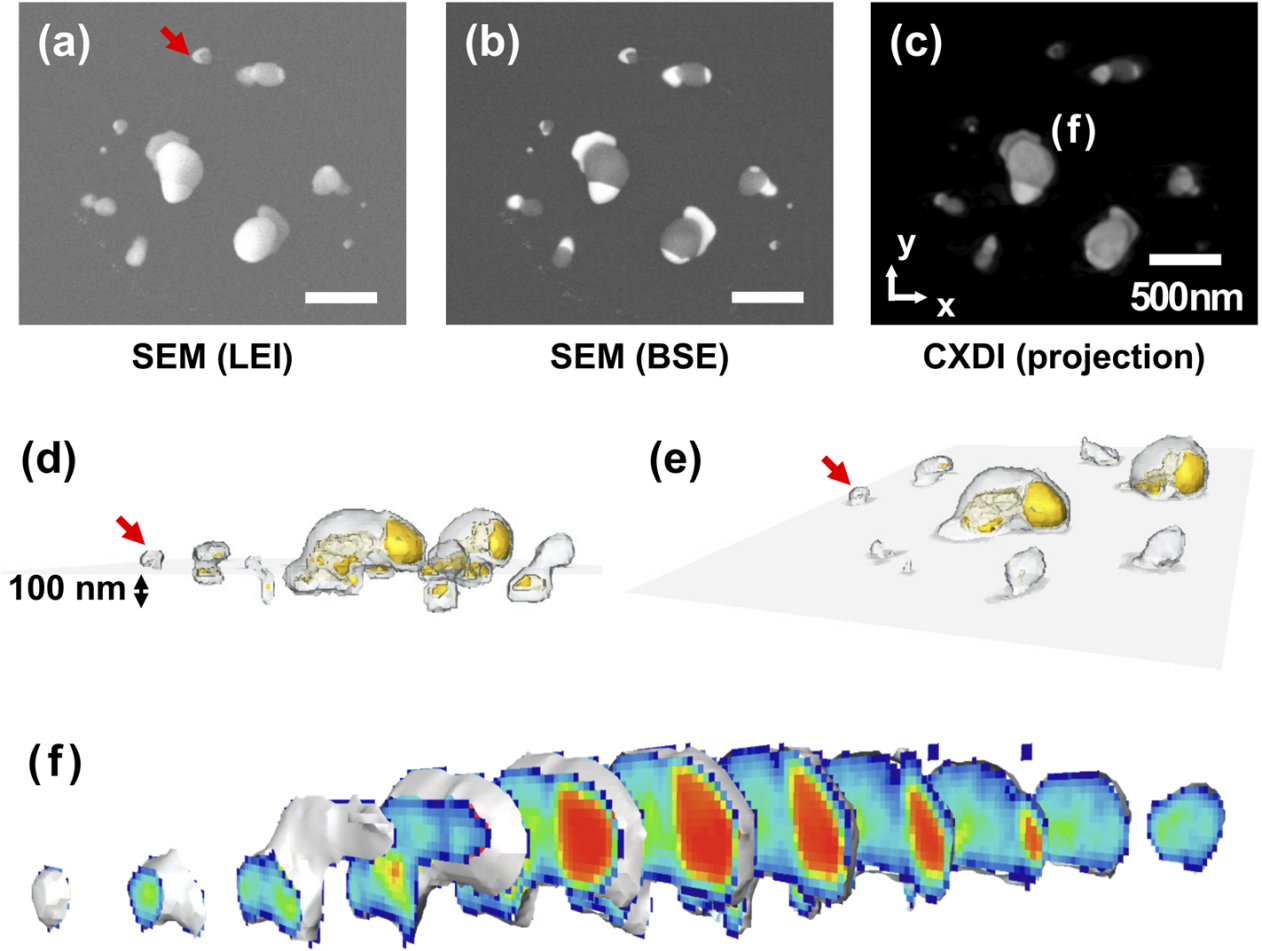
(a and b) SEM images of the phase separated AuNi nanoalloys in two different modes, (a) lower secondary electron image (LEI) and (b) back-scattered electron (BSE) modes. (c) Reconstructed real space CXDI image of the phase separated AuNi nanoalloys (projection image). (d and e) 3D rendering of the phase separated AuNi nanoalloys in two different viewpoints. Yellow colored area corresponds to higher electron density region, *e.g.*, Au_3_Ni. Almost all particles have foot-like structures although one particle indicated by red arrows does not have. (f) Sectioned images of one of AuNi particles, marked by (f) in (c), with 37.2 nm intervals in the *y*–*z* plane.

We performed the tomographic CXDI measurement on the phase separated AuNi nanoalloys including the eutectic AuSi compounds to investigate the internal compositions and foot-like structures non-destructively. The tomographic sample was first measured by SEM (see Fig. 1a and b) in two different modes, the lower secondary electron image (LEI) and the back-scattered electron (BSE) modes, since the former is more sensitive to morphology and the latter is more sensitive to elemental information. The series of 27 projected diffraction patterns of the AuNi nanoalloys including the eutectic AuSi compounds was measured at various incident angles ranging from −69.44° to 69.44° based on the equally sloped tomography (EST).^30^ The incident photon flux, *I*_0_, after the beam defining W pinhole was about 1.1 × 10^7^ photons per s per μm^2^ and diffraction patterns were collected for 20 minutes at each angle. Individual 2D projection images were reconstructed by using the hybrid input–output (HIO) algorithm.^31,32^ Firstly, 50 independent reconstructions with rectangular support whose size is calculated based on the speckle size at each angle were performed for 3000 iterations. The best 3 results that have the smallest reciprocal error value among all reconstructions were averaged, and the support was refined to exclude 0-density area. With the updated support, fresh 50 independent reconstructions were performed for 3000 iterations and again the best 3 results were averaged as a final image. Then the EST algorithm was applied on the 27 projections to reconstruct a 3D electron density image with the final voxel size of (12.4 nm)^3^. The total electron density, *N*_e_, of the nanoalloys is 1.04 × 10^11^ electrons which was estimated as follows:1
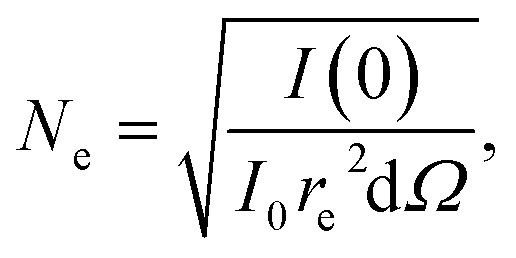
where *I*(0), *r*_e_, and d*Ω* are the number of diffracted X-ray photons to the central detector pixel, the electron scattering length, and the solid angle covered by the central pixel, respectively.^33,34^

Fig. 1c shows a projected CXDI image of the phase separated AuNi nanoalloys measured at 0° projection angle and Fig. 1d and e show sideview and bird's eye view of rendered images of the reconstructed 3D image. Almost all particles have foot-like structures which are embedded in the Si_3_N_4_ substrate but one particle indicated by red arrows in Fig. 1a–e does not. We focused on two big particles in Fig. 1d and e which have three different regions, yellow (head-like structures), whitish yellow (foot-like structures and connections between heads and feet), and white regions and each of them corresponds to Au_3_Ni, Au-rich AuSi, and Ni-rich NiSi respectively. More details about specific compositions and forming processes will be discussed in the following chapters. Fig. 1f shows a series of cross sectional images of one particle, marked as (f) in Fig. 1c, with 37.2 nm intervals. The internal electron density of the head-like structure is uniform while the foot-like structure and the connection between them are rather irregular.

**Fig. 2:**
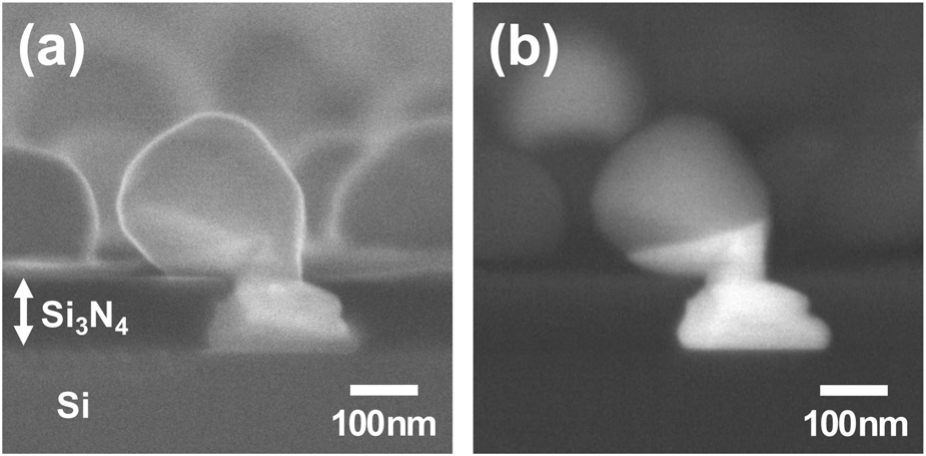
Cross-sectional SEM images of the phase separated AuNi nanoalloys in (a) LEI and (b) BSE modes. The sample was prepared by following the same procedure used for the tomography sample except substrate and the initial pattern.

For better understanding of the foot-like structures, we prepared another phase separated AuNi nanoalloy sample by following the same procedure used for the tomography sample except for the substrate and without pattern. Here, Ni–Au films were deposited on a 100 nm thick Si_3_N_4_ layer supported by a thick Si(100) substrate and annealed at 850 °C in vacuum environment (∼10^−6^ mbar) for 20 min. Cross-sectional SEM images in the LEI and the BSE modes are displayed in Fig. 2(a) and (b), respectively. In this case, Au atoms are fully moved to foot-like structure and formed Au_5_Si_2_ alloy since the reaction process of smaller particles is faster than that of bigger particles.

### 3.2 Time-series study on the eutectic process of the AuSi compounds

**Fig. 3:**
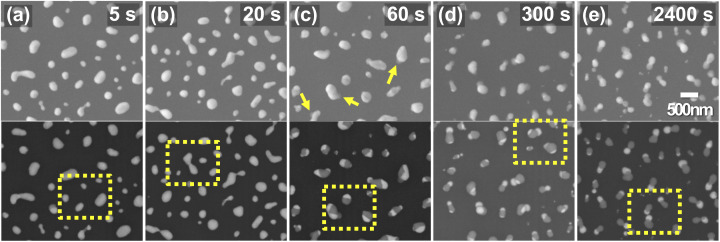
SEM images of a series of samples with different annealing times, (a) 5 s, (b) 20 s, (c) 60 s, (d) 300 s, and (e) 2400 s, in two different modes, the LEI (upper row) and the BSE (lower row) modes. Yellow arrows in (c) correspond to the Ni-rich foot-like structures which can be considered as initial interaction in between Ni–Au bilayer and Si_3_N_4_ layer. Yellow dotted rectangles in the BSE images are compared to our model shown in Fig. 8.

We first performed the SEM measurement including energy-dispersive X-ray (EDX) analysis on a series of samples with different annealing times (Fig. S1–S3). Fig. 3 shows representative SEM images of the time-series samples. The upper and the lower rows correspond to the images in the LEI and the BSE modes, respectively. Relatively bright area in the BSE images, *e.g.*, white area in Fig. 3c, represents Au-rich phase, and relatively dark region, *e.g.*, gray area in Fig. 3c, represents Ni-rich phase. The entire SEM images of the time-series samples can be found in the SI.

Isolated particles are formed through the solid state dewetting process, mainly grain boundary grooving^35^ in the early stage (Fig. 3a) and phase separations on a part of the nanoparticles are observed at around 20 s (Fig. 3b). Then, Ni-rich foot-like structure as well as clearly phase separated particles are formed as shown in Fig. 3c. Yellow arrows in Fig. 3c correspond to Ni-rich foot-like structures. As the annealing time increases, Au atoms move to the substrate along domain boundaries and form Au-rich foot-like structures while Ni atoms move to the other side.

In the BSE images, The Au-rich and Ni-rich foot-like structures can be distinguished. A certain area, several hundreds of square micrometers, of the BSE images were selected and the number of Au-rich and Ni-rich foot-like structures were counted. As the annealing time increases, the number of Ni-rich foot-like structures decreases while the number of Au-rich foot-like structures increases. See Fig. S4 in SI for more details. The same time-series samples but grown on 50 nm thick Si_3_N_4_ membrane windows were prepared (see SI, Fig. S3) and the same counting process was performed. It shows the probability plot of the samples grown on membrane windows and the trend is more clearly visible.

**Fig. 4:**
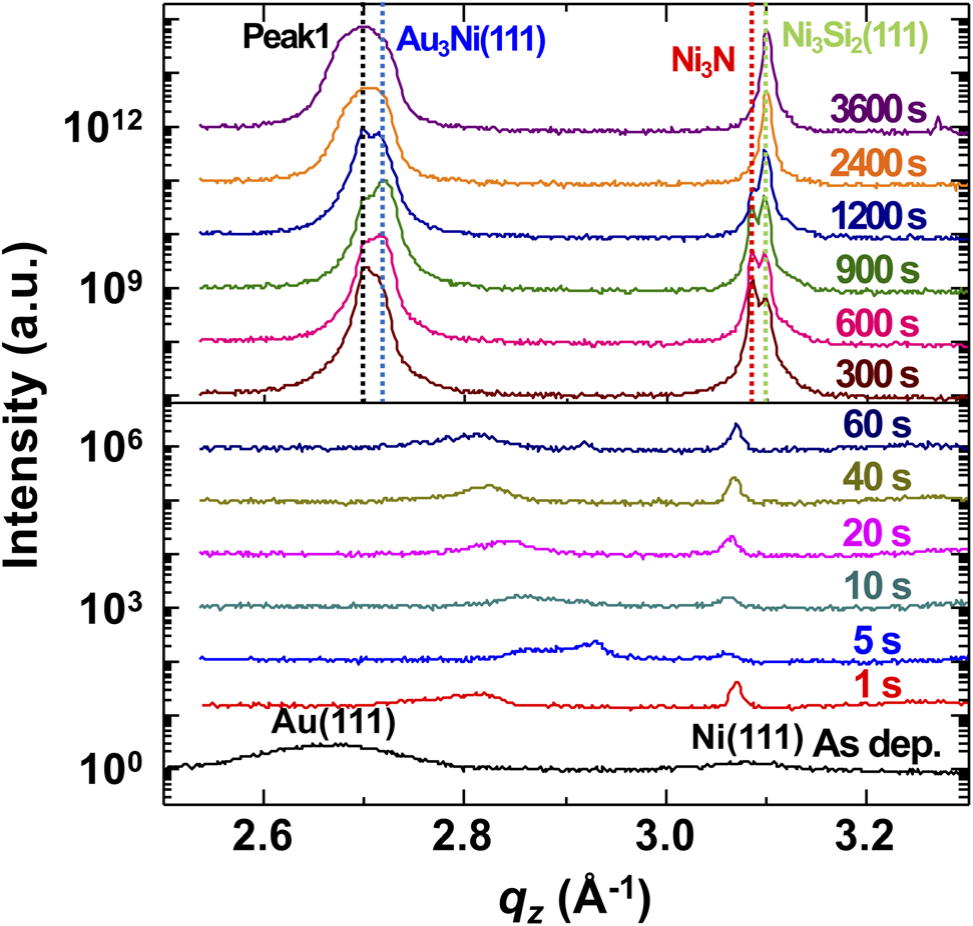
XRD profiles of the time-series samples around the Au(111) and Ni(111) bragg peaks. The atomic exchange at the interfaces and the solid state dewetting occur until 60 s. Four major peaks, marked as peak 1, Au_3_Ni(111), Ni_3_N, and Ni_3_Si_2_(111), are formed after 60 s. The peak 1 can be considered as NiN in the beginning and Au_5_Si_2_ in the end.

**Fig. 5:**
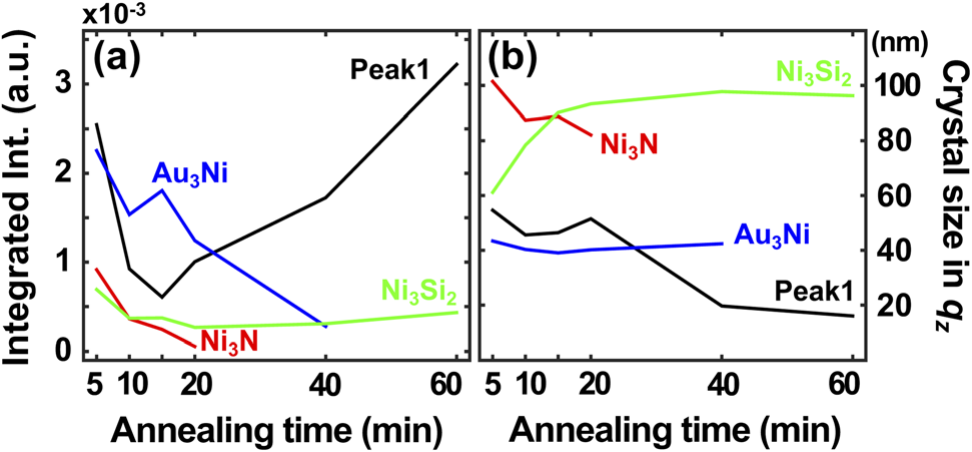
(a) Integrated intensities and (b) estimated crystal sizes in *q*_*z*_ direction calculated by the scherrer equation of the XRD profiles of the time-series samples. The crystal size corresponds to the depth direction (*z* axis) of the 3D CXDI image. The integrated intensities of Au_3_Ni and Ni_3_N decrease gradually and disappear at around 20 min and 40 min. The integrated area of the peak 1 also decreases until 15 min, which is relevant to NiN peak, and increases again afterward, which is relevant to Au_5_Si_2_ peak. In the end, metastable Au_5_Si_2_ and Ni_3_Si_2_ are formed.

We performed the XRD measurement on the time-series samples to understand atomic behavior during the solid state dewetting and agglomeration processes. Fig. 4 shows a series of XRD profiles of the time-series samples, and Fig. 5 shows integrated intensities (a) and estimated crystal sizes in *q*_*z*_ direction calculated from the XRD profiles (b). In the early stages, broad peaks are moving around near Au(111) and Ni(111) peaks since there happened the atomic exchange at the interfaces, Si_3_N_4_–Ni and Ni–Au, and the solid state dewetting process near the boundary and defects. Two peaks, around 2.81 and 3.07 Å^−1^, in 1s XRD data correspond to Au-rich and Ni-rich phases. Four intense peaks, marked as peak 1, Au_3_Ni(111), Ni_3_N, and Ni_3_Si_2_(111), are formed after 60 s (1 min). The peak 1 can be considered as NiN at the initial stage and Au_5_Si_2_ at the later stage. The peak 1 and Ni_3_N peak keep decreasing until 1200 s (20 min) and Ni_3_N peak vanishes at 2400 s (40 min) since the N atoms are evaporated by forming N_2_.^36^ The integrated area of the peak 1 is also decreasing until 900 s (15 min), which is relevant to NiN peak, and increased afterward, which is relevant to Au_5_Si_2_ peak.^37–40^ A grain size of the Au_3_Ni in *q*_*z*_ direction remains the same although its integrated area keeps decreasing and disappears after 2400 s (40 min), which can be the evidence of grain boundary diffusion of Au atoms. The Ni and Au atoms from the Au_3_Ni and NiN gradually combined with Si atoms and form Au_5_Si_2_ and Ni_3_Si_2_.

There are two major steps in the aforementioned processes: (1) reaction between Ni and Si_3_N_4_ and (2) movement of Au atoms to the substrate side. A clue of the former can be found from other studies related to Co annealing process^36,41^ since the band structure of Ni is similar to that of Co.^42^ Based on ref. 37, 40 and 41, we can assume that the Ni atoms weaken Si–N bonds and form strong p–d bonds with Si resulting in formation of nickel silicide and nickel nitride even at relatively low temperature.^36^ The latter can be explained by atomic diffusion of Au & Ni atoms and the eutectic behavior of AuSi compound. Au atoms tend to move below Ni layer while Ni atoms move the opposite direction since it is energetically more favorable.^19,20^ Au atoms favour grain boundary diffusion whereas Ni atoms favour lattice diffusion because of the big lattice mismatch and different lattice diffusion coefficient. The eutectic temperature of AuSi compound is 363 ± 3 °C which makes Au atoms more mobile and the diffusion process more efficient.^37^

### 3.3 Electron density evaluation and a model system for the AuSi eutectic behavior

**Fig. 6:**
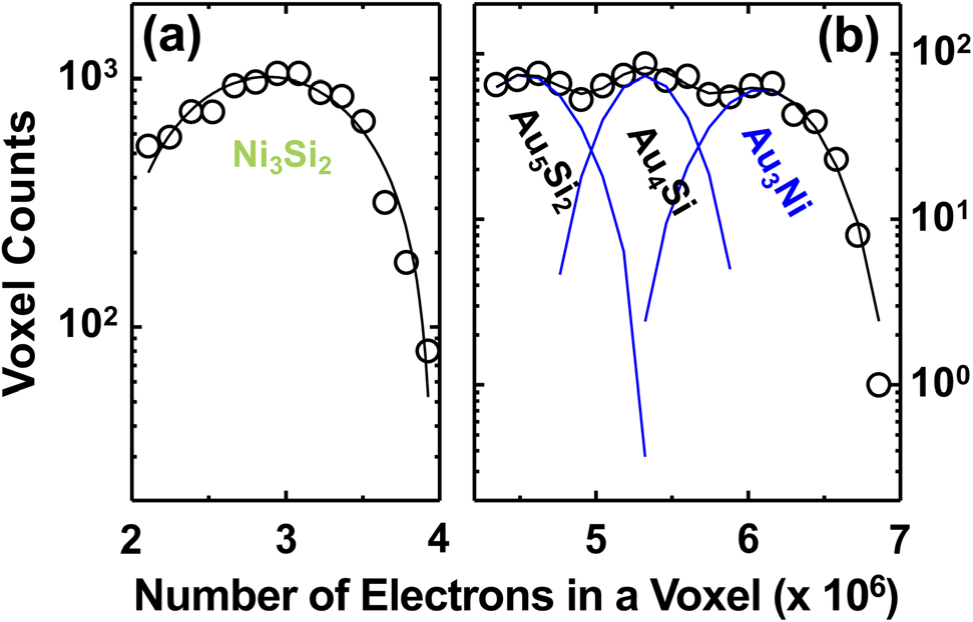
Histograms of the reconstructed 3D CXDI image in (a) Ni-rich and (b) Au-rich electron density regions (single and multiple-peak gaussian fitting were applied to each region separately). There are four major electron densities, 2.9 × 10^6^, 4.5 × 10^6^, 5.3 × 10^6^, and 6.1 × 10^6^, which correspond to Ni_3_Si_2_, Au_5_Si_2_, Au_4_Si, and Au_3_Ni respectively.

Fig. 6a and (b) show histogram analyses on the electrons in voxels of the reconstructed 3D CXDI image in Ni-rich and Au-rich electron density regions respectively. A more detailed explanation of the electron density calculations can be found in the SI, Fig. S6 and Table S1. With these, the calculated electron density of Au_3_Ni, 6.1 × 10^6^ e per voxel, obtained from the histogram analyses is ∼25% lower than the expected electron density, 8.1 × 10^6^ e per voxel. The absolute electron density values are rather inaccurate since the substrate materials were contributed to the electron density calculation, *e.g.*, foot-like structures in Fig. 1d–f have electron density values while neighbouring Si_3_N_4_ substrate does not have. Also, nitrogen atoms in NiN compound gradually evaporated by forming N_2_. These factors may affect the calculated electron density values. Nevertheless, the ratio of the electron densities of Ni_3_Si_2_, Au_5_Si_2_, Au_4_Si, and Au_3_Ni matches well with our histogram analysis, 1 : 1.6 : 1.8 : 2.1 (see SI, Table S1) and the XRD result. The width of Gaussian curves in Fig. 6, especially Ni_3_Si_2_ peak, is a bit broad since the atomic diffusion is occurring actively at this moment.

**Fig. 7:**
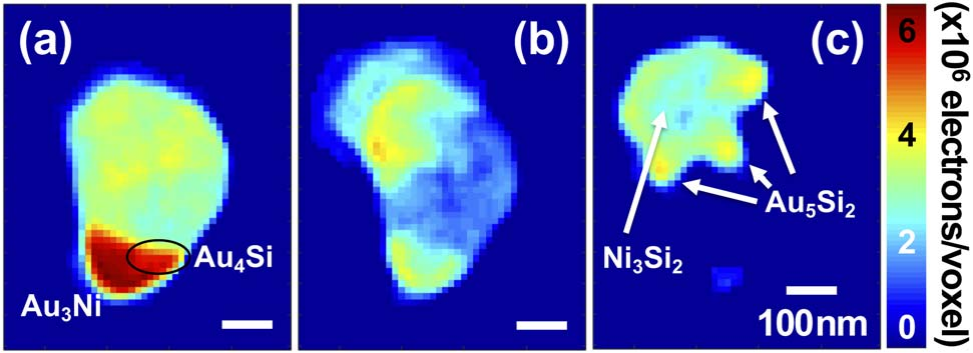
Three representative sectioned images of the AuNi particle, marked by (f) in Fig. 1(c), cut in the x-y plane. (a) 50 nm above, (b) near, and (c) 50 nm beneath the Si_3_N_4_ membrane surface.

Fig. 7 shows sectioned electron density images in the direction of the surface normal of the AuNi particle, marked by (f) in Fig. 1c. A complete set of the sectioned electron density images is available in the SI, Fig. S7. The Au-rich region, red coloured area in Fig. 7a, consists of two major electron density regions, Au_3_Ni and Au_4_Si (black ellipse) phases. The Au_4_Si alloy, approximately the eutectic composition (18.6 ± 0.5 at% of Si), is formed near the interface in between Au-rich and Ni-rich regions which indicates that the movement of Au atoms are mainly triggered by the eutectic behavior of AuSi alloys and happens along the interface (grain boundary). The XRD result related to Au_3_Ni peak showed that its grain size in *q*_*z*_ direction, the depth direction (z axis) of the 3D CXDI image, stayed the same until it is fully dissolved by Si atoms, which is consistent with the electron density evaluation. Fig. 7c shows a detailed foot-like structure located inside Si_3_Ni_4_ membrane. Boundaries of the foot-like structure whose electron density is close to Au_5_Si_2_ have higher electron density than inside and it is also an evidence of the grain boundary diffusion of Au atoms.

**Fig. 8:**
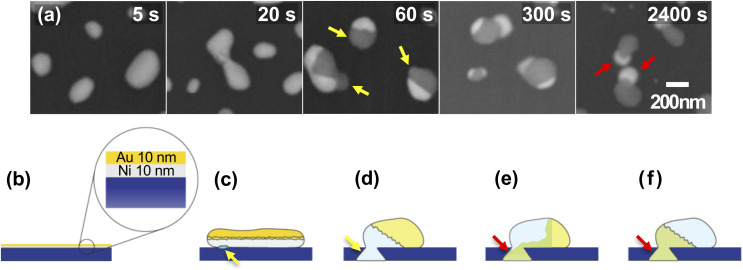
A model for the thermal annealing process of a Ni/Au bilayer film on Si_3_N_4_ substrate. (a) The representative SEM images in the BSE mode which correspond to yellow dotted rectangles in Fig. 3. (b) A Ni/Au bilayer film before the annealing process. (c) Shortly after the annealing process starts. AuNi bilayer films separate into islands that are composed of a mixture of Au-rich and Ni-rich phases. (d) The phase separated particle with Ni-rich foot-like structure is formed. (e) Au atoms from the head-like structure move to the substrate continuously and Au-rich foot-like structure is formed. (f) Final state of the reaction. All the Au atoms move to the substrate side and Ni atoms move to the other side. As a result, metastable Au_5_Si_2_ and Ni_3_Si_2_ compounds are formed. (c–f) Correspond to 5, 60, 300, and 2400 s SEM images in (a).

Based on our studies, the thermal annealing process in a Ni/Au bilayer thin film on a Si_3_N_4_ substrate can be simplified to 5 steps: 1. As-deposited Ni/Au bilayer film, 2. Dewetting into islands composed of mixture of Au-rich and Ni-rich phases, 3. Formation of the phase separated Ni-rich, mostly Ni_3_Si_2_, foot-like structure and Au-rich, mostly Au_3_Ni, head-like structure, 4. Movement of Au atoms to form Au-rich, mostly Au_5_Si_2_, foot-like structure mainly triggered by the formation of the eutectic AuSi compound, and 5. Formation of metastable Au_5_Si_2_ foot-like structure and Ni_3_Si_2_ compound in the other side. The simplified 5 steps are illustrated in Fig. 8b–f. To confirm our model, we compared them with the SEM images of the time-series samples as shown in Fig. 8a. Fig. 8c–f correspond to 5, 60, 300, and 2400 s SEM images in Fig. 8a. They are quite consistent and the detailed processes were confirmed by the tomographic CXDI and the XRD measurements.

## 4. Conclusions

We have successfully visualized the formation of a eutectic AuSi compound in 3D during the solid state dewetting and agglomeration in a Ni–Au bilayer thin film grown on a Si_3_N_4_ membrane window. In the early stage of the reaction, Au-rich and Ni-rich nanoparticles are formed by the dewetting and agglomeration processes. Then, the catalytic action of Ni atoms weakens Si–N bonds of the Si_3_N_4_ substrate and Ni atoms form strong p-d bonds with Si atoms resulting in the formation of NiSi and NiN alloys. As the annealing time increases, nitrogen atoms in NiN are gradually evaporated by forming N_2_ since it is energetically more favorable. Also, NiSi grains make an interface with Au_3_Ni alloys which results in the formation of the eutectic AuSi compound and it activates the grain boundary diffusion of Au atoms and boost up the whole reaction processes. In the last stage of the reaction, metastable Au_5_Si_2_ foot-like structures inside and near the Si_3_N_4_ substrate and Ni_3_Si_2_ head-like structures are formed.

The spatiotemporal resolution of the 3D CXDI method is not sufficient enough to make a 3D movie of the forming process of the nanoscale eutectic AuSi compounds, but the non-destructive 3D imaging capability of the CXDI method allows us to investigate the detailed chemical reaction in between the AuNi thin film and Si_3_N_4_ substrate. We believe that this work paths the way for a better understanding of solid-state dewetting and agglomeration processes of metallic thin films, and will provide a clue to fabricate high quality devices and nanoparticles.

## Author contributions

Y. Kim, C. Kim and D. Y. Noh conceived the experiment. The XRD and CXDI experiments were performed by all authors. The XRD and CXDI data analysis were performed by Y. Kim and C. Kim with support from S. Y. Lee, H. C. Kang, and D. Y. Noh. The manuscript was written by Y. Kim and C. Kim with input from all authors.

## Conflicts of interest

There are no conflicts to declare.

## Supplementary Material

RA-015-D5RA04389A-s001

## Data Availability

The CXDI data that support the findings of this study are openly available in Zenodo, at https://doi.org/10.5281/zenodo.16357335, reference number 10.5281/zenodo.16357335. Derived data supporting the findings of this study are available from the corresponding author upon reasonable request. Supplementary information is available. See DOI: https://doi.org/10.1039/d5ra04389a.

## References

[cit1] Wang J., Lu X.-G., Sundman B., Su X. (2005). Calphad.

[cit2] Sivertsen J., Wert C. (1959). Acta Metall..

[cit3] Koch F. B., Sivertsen J., Sundahl R. (1961). Acta Metall..

[cit4] Wolverton C., Zunger A. (1997). Comput. Mater. Sci..

[cit5] Lee S., Jang H., Noh D., Kang H. (2007). Appl. Phys. Lett..

[cit6] Gilroy K. D., Ruditskiy A., Peng H.-C., Qin D., Xia Y. (2016). Chem. Rev..

[cit7] Li Q., Xue S., Wang J., Shao S., Kwong A. H., Giwa A., Fan Z., Liu Y., Qi Z., Ding J., Wang H., Greer J. R., Wang H., Zhang X. (2018). Adv. Mater..

[cit8] Hrauda N., Zhang J., Wintersberger E., Etzelstorfer T., Mandl B., Stangl J., Carbone D., Holý V., Jovanović V., Biasotto C., Nanver L. K., Moers J., Grützmacher D., Bauer G. (2011). Nano Lett..

[cit9] Huang Z., Bartels M., Xu R., Osterhoff M., Kalbfleisch S., Sprung M., Suzuki A., Takahashi Y., Blanton T. N., Salditt T., Miao J. (2015). Nat. Mater..

[cit10] Miao J., Ishikawa T., Robinson I. K., Murnane M. M. (2015). Science.

[cit11] Chapman H. N., Nugent K. A. (2010). Nat. Photonics.

[cit12] Ulvestad A., Welland M., Collins S., Harder R., Maxey E., Wingert J., Singer A., Hy S., Mulvaney P., Zapol P., Shpyrko O. G. (2015). Nat. Commun..

[cit13] Mastropietro F., Godard P., Burghammer M., Chevallard C., Daillant J., Duboisset J., Allain M., Guenoun P., Nouet J., Chamard V. (2017). Nat. Mater..

[cit14] Donnelly C., Guizar-Sicairos M., Scagnoli V., Gliga S., Holler M., Raabe J., Heyderman L. J. (2017). Nature.

[cit15] Kim C., Chamard V., Hallmann J., Roth T., Lu W., Boesenberg U., Zozulya A., Leake S., Madsen A. (2018). Phys. Rev. Lett..

[cit16] Chen N.-J., Yeh C.-H., Cao H.-Y., Chen N.-C., Chen C.-J., Chen C.-Y., Tsai Y.-W., Lin J.-M., Huang Y.-S., Hsiao C.-N., Chen C.-C. (2025). Synchrotron Radiat..

[cit17] Ayyer K., Xavier P. L., Bielecki J., Shen Z., Daurer B. J., Samanta A. K., Awel S., Bean R., Barty A., Bergemann M., Ekeberg T., Estillore A. D., Fangohr H., Giewekemeyer K., Hunter M. S., Karnevskiy M., Kirian R. A., Kirkwood H., Kim Y., Koliyadu J., Lange H., Letrun R., Lübke J., Michelat T., Morgan A. J., Roth N., Sato T., Sikorski M., Schulz F., Spence J. C. H., Vagovic P., Wollweber T., Worbs L., Yefanov O., Zhuang Y., Maia F. R. N. C., Horke D. A., Küpper J., Loh N. D., Mancuso A. P., Chapman H. N. (2020). Optica.

[cit18] E J., Kim Y., Bielecki J., Sikorski M., de Wijn R., Fortmann-Grote C., Sztuk-Dambietz J., Koliyadu J., Letrun R., Kirkwood H., Sato T., Bean R., Mancuso A. P., Kim C. (2022). Struct. Dyn..

[cit19] Cen X., Thron A. M., van Benthem K. (2017). Acta Mater..

[cit20] Cen X., Thron A. M., Zhang X., van Benthem K. (2017). Ultramicroscopy.

[cit21] Kaplan W. D., Chatain D., Wynblatt P., Carter W. C. (2013). J. Mater. Sci..

[cit22] Thompson C. V. (2012). Annu. Rev. Mater. Res..

[cit23] Cahn J. W., Hilliard J. E. (1958). J. Chem. Phys..

[cit24] Wang D., Schaaf P. (2012). Mater. Lett..

[cit25] Kim D., Giermann A. L., Thompson C. V. (2009). Appl. Phys. Lett..

[cit26] Deduytsche D., Detavernier C., Van Meirhaeghe R., Lavoie C. (2005). J. Appl. Phys..

[cit27] Kim Y., Kim J., Ahn K. W., Noh D. Y., Kim C., Kang H. C., Lee H. C., Yu C.-J. (2017). J. Korean Phys. Soc..

[cit28] Yu C.-J., Lee H. C., Kim C., Cha W., Carnis J., Kim Y., Noh D. Y., Kim H. (2014). J. Synchrotron Radiat..

[cit29] Kim Y., Kim C., Ahn K., Choi J., Lee S. Y., Kang H. C., Noh D. Y. (2020). J. Synchrotron Radiat..

[cit30] Miao J., Förster F., Levi O. (2005). Phys. Rev. B:Condens. Matter Mater. Phys..

[cit31] Fienup J. R. (1978). Opt. Lett..

[cit32] Fienup J. R. (1982). Appl. Opt..

[cit33] Miao J., Amonette J. E., Nishino Y., Ishikawa T., Hodgson K. O. (2003). Phys. Rev. B:Condens. Matter Mater. Phys..

[cit34] Kim Y., Kim C., Kwon O. Y., Nam D., Kim S. S., Park J. H., Kim S., Gallagher-Jones M., Kohmura Y., Ishikawa T., Song C., Noh D. Y. (2017). Sci. Rep..

[cit35] Amram D., Klinger L., Gazit N., Gluska H., Rabkin E. (2014). Acta Mater..

[cit36] Nguyen T., Ho H. L., Kotecki D. E., Nguyen T. D. (1993). J. Mater. Res..

[cit37] Okamoto H., Massalski T. (1983). Bull. Alloy Phase Diagrams.

[cit38] Ghatak J., Bhatta M. U., Sundaravel B., Nair K., Liou S.-C., Chen C.-H., Wang Y.-L., Satyam P. (2008). Nanotechnology.

[cit39] Hübner J.-M., Bierman B. C., Wallenberg R., Fredrickson D. C. (2022). J. Am. Chem. Soc..

[cit40] Aballe L., Gregoratti L., Barinov A., Kiskinova M., Clausen T., Gangopadhyay S., Falta J. (2004). Appl. Phys. Lett..

[cit41] Weaver J. H., Franciosi A., Moruzzi V. L. (1984). Phys. Rev. B:Condens. Matter Mater. Phys..

[cit42] Lambrecht W. R., Christensen N. E., Blöchl P. (1987). Phys. Rev. B:Condens. Matter Mater. Phys..

