# Supplementary material for: Nanoscale Au–Si eutectic mixtures formed by dewetting of a Au–Ni film on Si3N4
Source: RSC Adv. 2025 Oct 31;15(49):41917–23. doi: 10.1039/d5ra04389a (PMC12576887; doi:10.1039/d5ra04389a)
Supplement: RA-015-D5RA04389A-s001 [file RA-015-D5RA04389A-s001.pdf]

Supplementary Information (SI) for RSC Advances.
This journal is © The Royal Society of Chemistry 2025

Supplementary Information

Nanoscale Au-Si Eutectic Mixtures Formed by Dewetting of a Au-Ni film on Si_3N_4

Yoonhee Kim,*[a] Kang Woo Ahn,[b‡] Su Yong Lee,[c] Jin Woo Kim,[c] Hyon Chol Kang,[d] Chan Kim,*[a] and Do Young Noh*[b]

[a] *European X-ray Free-Electron Laser Facility, Holzkoppel 4, Schenefeld 22869, Germany. E-mails: yoonhee.kim@xfel.eu, chan.kim@xfel.eu*

[b] *Department of Physics and Photon Science and School of Materials Science and Engineering, Gwangju Institute of Science and Technology, Gwangju 61005, Korea. E-mail: dynoh@gist.ac.kr*

‡ Present address: Pohang Accelerator Laboratory, Pohang 37673, Korea

[c] *Pohang Accelerator Laboratory, Pohang 37673, Korea*

[d] *Department of Materials Science and Engineering, Chosun University, Gwangju 61452, Korea*

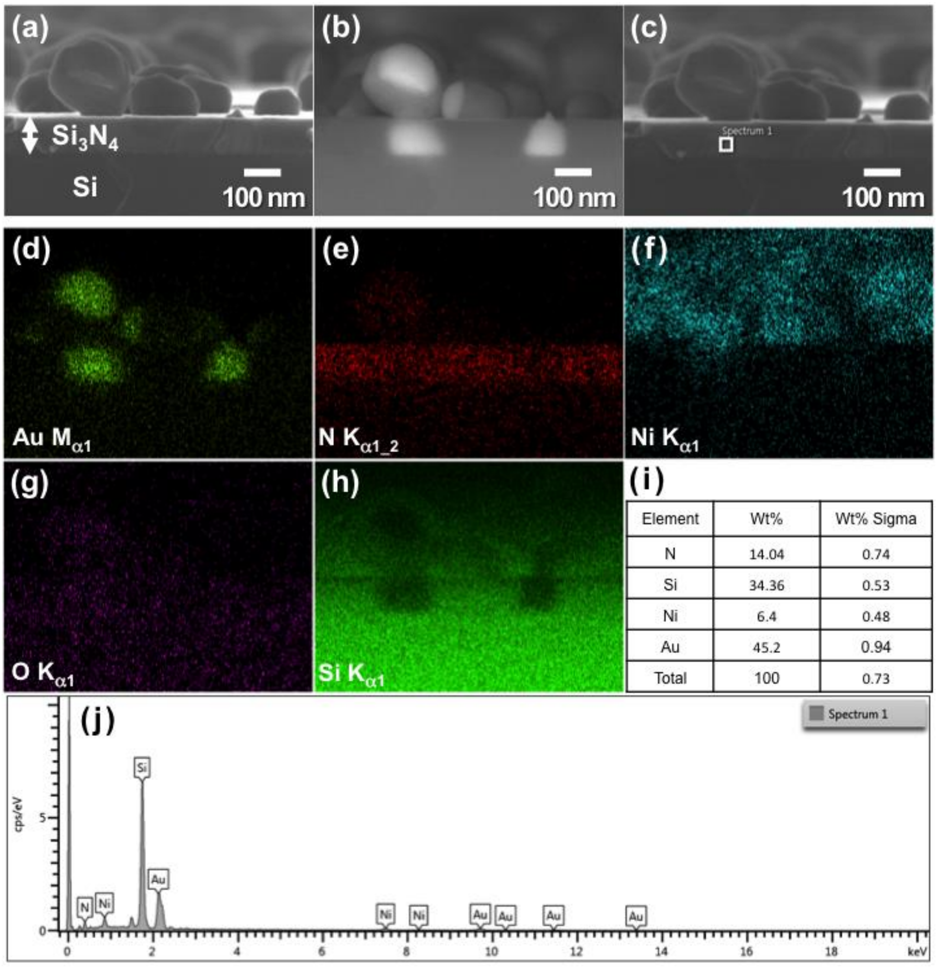

Element	Wt%	Wt% Sigma
N	14.04	0.74
Si	34.36	0.53
Ni	6.4	0.48
Au	45.2	0.94
Total	100	0.73

Fig. S1 (a-c) Cross-sectional SEM images of the phase separated AuNi nanoalloys in two different modes, (a,c) lower secondary electron image (LEI) and (b) back-scattered electron (BSE) modes. The sample was prepared by following exactly the same procedure used for the tomography sample except substrate and the initial pattern. Square box in (c) was selected for a spectrum measurement as shown in (i) and (j). (d-h) Results of energy dispersive X-ray spectroscopy (EDX) measurement, (d) Au $M_{\alpha1}$, (e) N $K_{\alpha1\_2}$ (f) Ni $K_{\alpha1}$, (g) O $K_{\alpha1}$, and (h) Si $K_{\alpha1}$ emission lines. (i,j) Quantitative results of the EDX spectrum analysis measured from the white square box in (c).

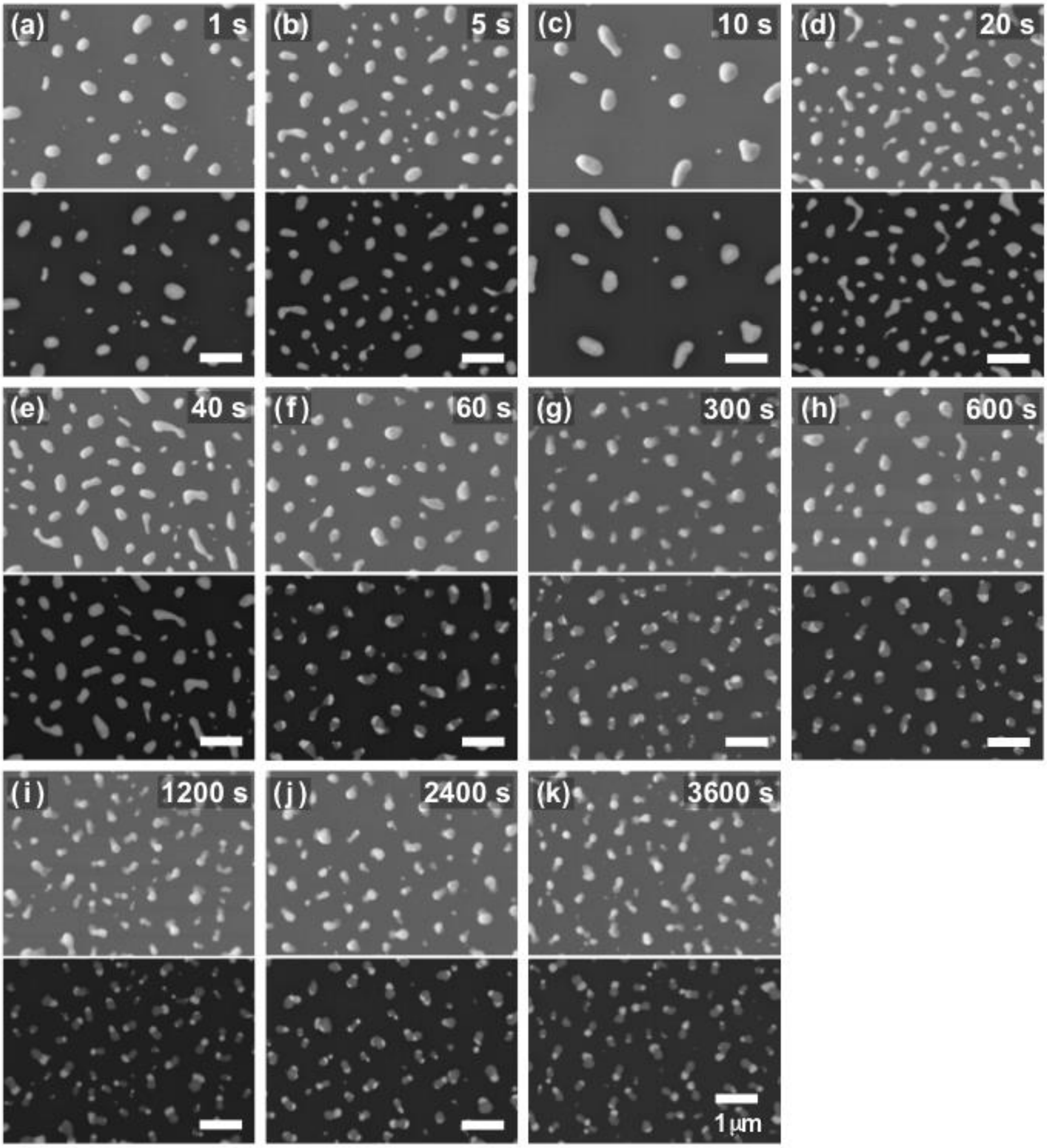

Fig. S2 SEM images of a series of samples with different annealing times, (a) 1 s, (b) 5 s, (c) 10 s, (d) 20 s, (e) 40 s, (f) 60 s, (g) 300 s, (h) 600 s, (i) 1200 s, (j) 2400 s, and (k) 3600 s, in two different modes, the LEI (upper row) and the BSE (lower row) modes. Ni/Au bilayer thin film, each of 10 nm, is deposited on a 100 nm thick Si_3N_4 substrate supported by a thick Si(100) in this case. Selected SEM images, 5 s, 20 s, 60 s, 300 s, and 2400 s, are shown in main Fig. 3.

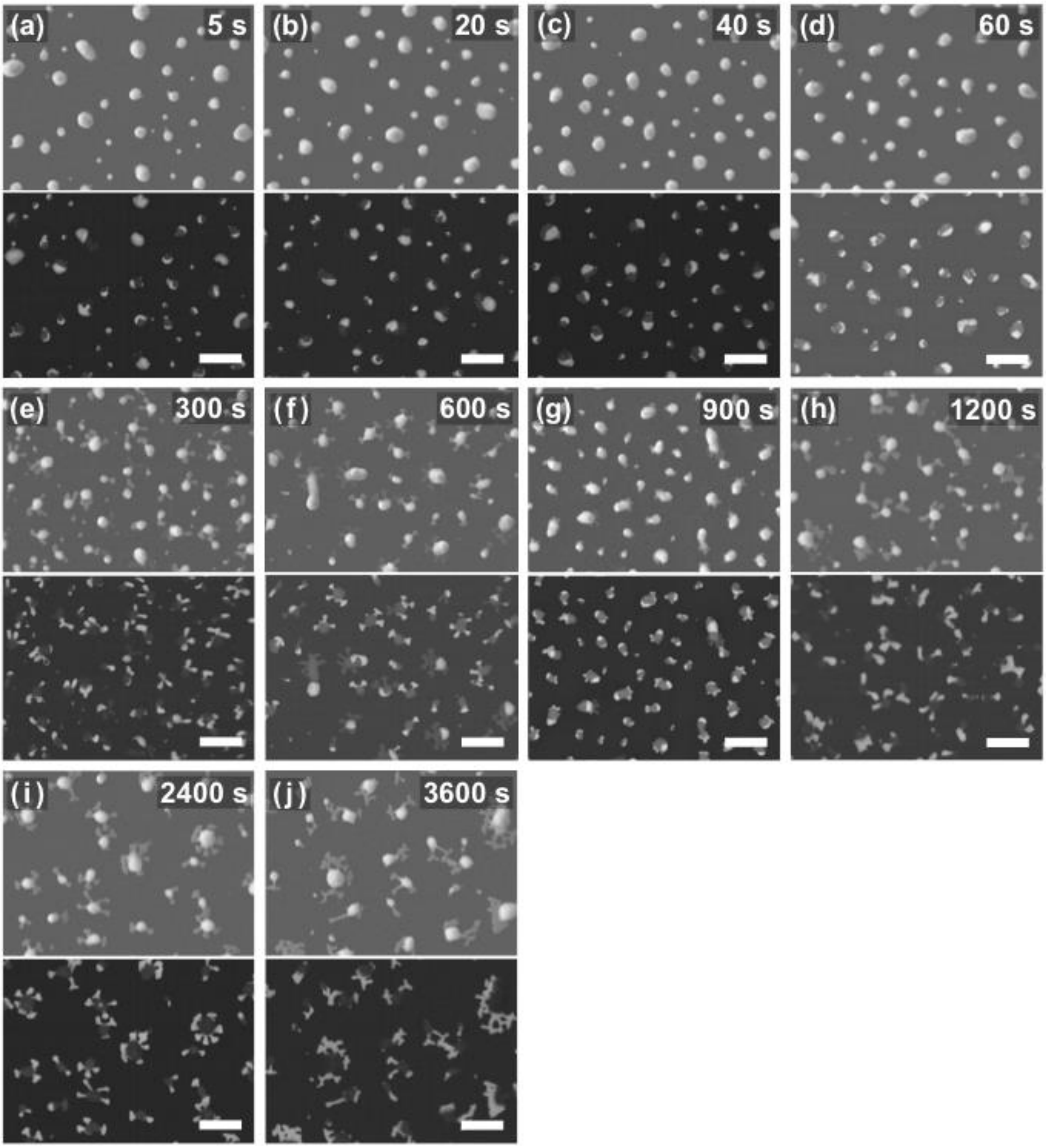

Fig. S3 SEM images of a series of samples with different annealing times, (a) 5 s, (b) 20 s, (c) 40 s, (d) 60 s, (e) 300 s, (f) 600 s, (g) 900 s, (h) 1200 s, (i) 2400 s, and (j) 3600 s, in two different modes, the LEI (upper row) and the BSE (lower row) modes. Ni/Au bilayer thin film, each of 10 nm, is deposited on a 50 nm thick Si_3N_4 membrane windows in this case. Standalone thin membrane may have more defects and it may cause more foot-like structures.

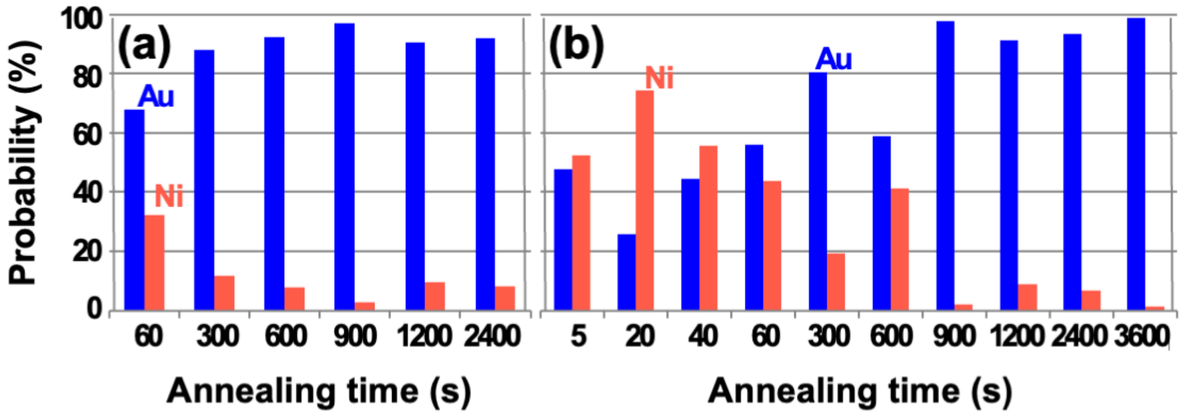

Fig. S4 Probability of having Au-rich and Ni-rich foot-like structures versus annealing time. (a) Ni/Au bilayer thin films, each of 10 nm, are deposited on 100 nm thick Si_3N_4 layer supported by thick Si(100) substrate which correspond to Fig. 3. (b) Ni/Au bilayer thin films, each of 10 nm, are deposited on 50 nm thick Si_3N_4 membrane windows.

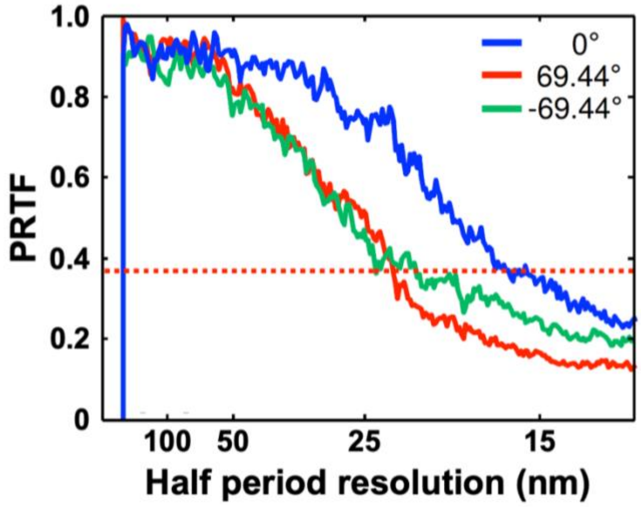

Fig. S5 Phase retrieval transfer function (PRTF) evaluations[1,2] of two-dimensional projection CXDI results at three different angles, 0° and ±69.44°. PRTF results were compared with 1/e level (red dotted horizontal line) and the achieved image resolution is around 20 nm in the half-period resolution. The final voxel size of the reconstructed 3D image was $(12.4\ nm)^3$.

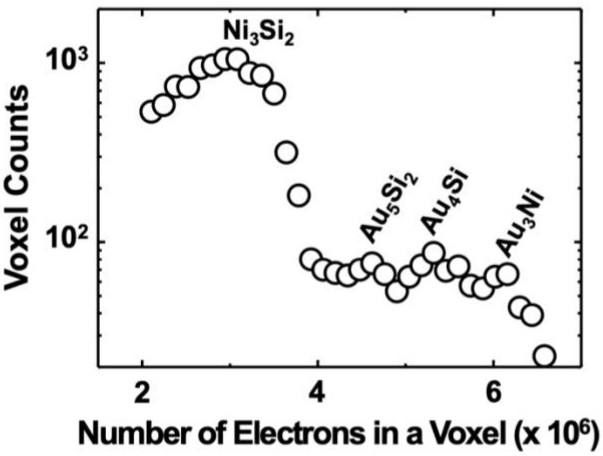

Fig. S6 Histogram of the reconstructed 3D CXDI image. The result of the Gaussian fitting was shown in main Fig. 6. Ni_3Si_2, Au_5Si_2, Au_4Si, and Au_3Ni are selected since three of them, Ni_3Si_2, Au_5Si_2, and Au_3Ni, are confirmed by XRD analysis and one of them, Au_4Si, is close to the eutectic composition, 18.6±0.5 At% of Si. All the peaks, especially Ni_3Si_2 peak, have broad widths since the atomic diffusion is occurring actively at this moment.

Table S1: Estimated electron densities of the relevant elements, alloys, and compounds in this study.

Material	Density (g/cm^3)	Molar mass (g/mol)	Lattice parameters (Å)	e density (e/nm^3)	Comments
Au	19.32	196.967	4.065	4665	FCC
Ni	8.902	58.693	3.499	2557	FCC
Au_3Ni	-	-	3.957	4277[b]	FCC
a-Au_4Si[a]	15.43[3]	162.936	-	3762[b]	0.0947 mol/cm^3
a-Au_7Si_3[a]	13.73[3]	146.375	-	3360[b]	0.0938 mol/cm^3
Ni_3Si_2	-	-	12.229, 10.805, 6.924[4]	1959[b]	80 atoms/unit cell
Si_3N_4	3.44	140.2833	-	136	-

[a] Au_4Si and Au_7Si_3 are chosen since their compositions are close to the eutectic composition (18.6±0.5 At% of Si) and Au_5Si_2, and their density and molar mass information are available in literature; [b] The ratio of the electron densities of Ni_3Si_2, a-Au_7Si_3, a-Au_4Si, and Au_3Ni is 1 : 1.7 : 1.9 : 2.2 which fit to our histogram analysis, as shown in main Fig. 6, with less than 5 % mismatch.

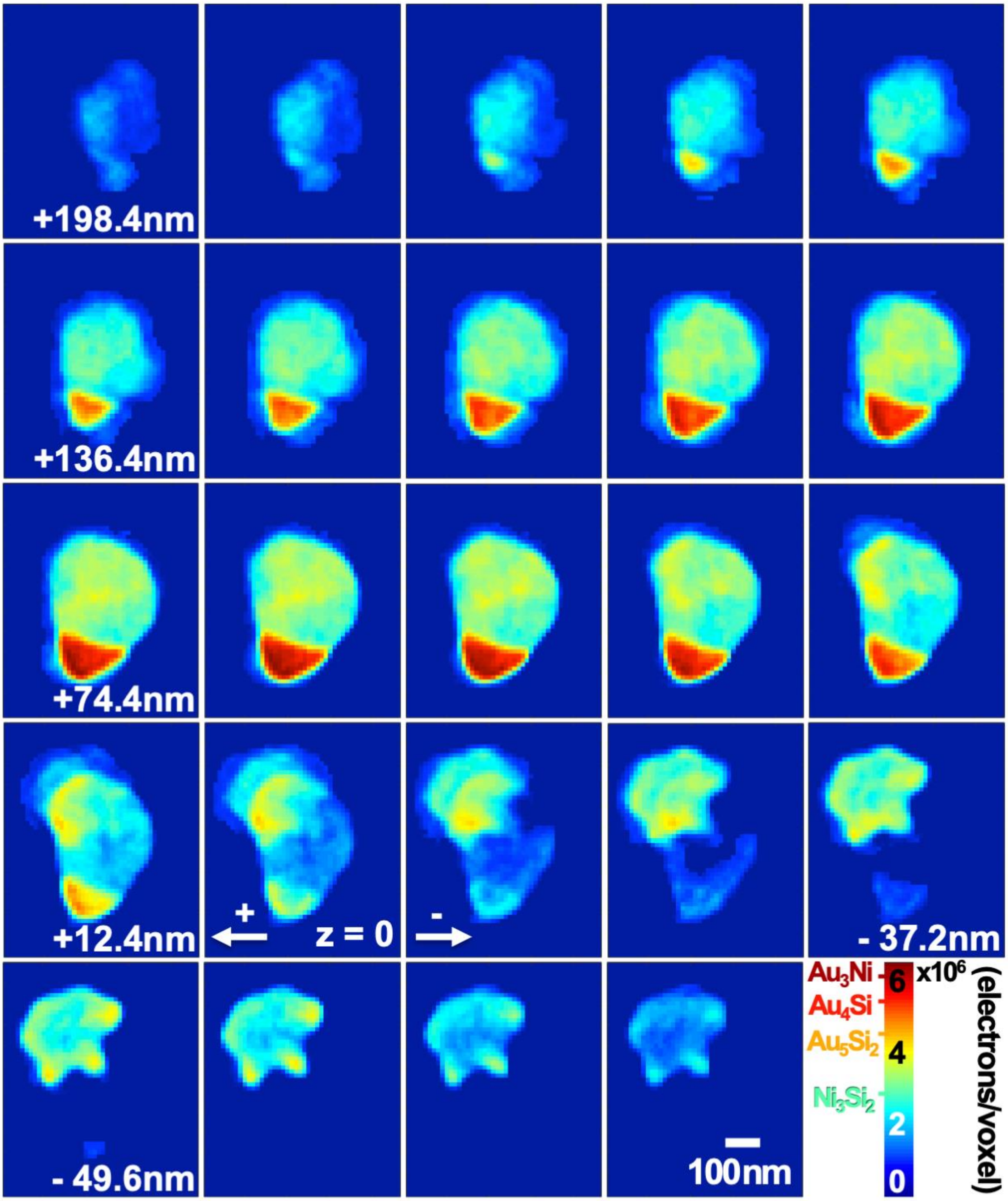

Fig. S7 A series of sectioned electron density maps of the nanoalloy particle, marked by (f) in main Fig. 1(c), cut in x-y plane. z = 0 corresponds to near the Si_3N_4 membrane surface and positive (negative) value means above (beneath) the surface.

References

1 H. N. Chapman, A. Barty, S. Marchesini, A. Noy, S. P. Hau-Riege, C. Cui, M. R. Howells, R. Rosen, H. He, J. C. H. Spence, U. Weierstall, T. Beetz and C. Jacobsen, *J. Opt. Soc. Am. A*, 2006, **23**, 1179-1200.
2 Y. Kim, C. Kim, O. Y. Kwon, D. Nam, S. S. Kim, J. H. Park, S. Kim, M. Gallagher-Jones, Y. Kohmura, T. Ishikawa, C. Song, G. Tae and D. Y. Noh, *Sci. Rep.*, 2017, **7**, 1850.
3 Ph. Mangin, G. Marchal, C. Mourey and Chr. Janot, *Phys. Rev. B*, 1980, **22**(8), 3047-3056.
4 G. Pilström, *Acta Chem. Scand.*, 1961, **15**, 893-902.
